# Calixpyrrole Derivatives: “Multi Hydrogen Bond” Catalysts for γ-Butenolide Synthesis

**DOI:** 10.3390/molecules14072594

**Published:** 2009-07-15

**Authors:** Grazia Cafeo, Margherita De Rosa, Franz H. Kohnke, Annunziata Soriente, Carmen Talotta, Luca Valenti

**Affiliations:** 1 Department of Chemistry, University of Salerno, via Ponte don Melillo, 84084 Fisciano (SA), Italy; E-mail: maderosa@unisa.it (M-D.R.); 2 Department of Organic and Biological Chemistry, University of Messina, Salita Sperone 31, 98166 Messina, Italy; E-mails: ella@isengard.unime.it (G.C.), luca.val2@libero.it (L.V.)

**Keywords:** calixpyrroles, diastereoselective synthesis, organocatalysis, aldol addition, 2-Trimethyl-silyloxyfuran

## Abstract

Calix[4]pyrrole (**1**)**,** calix[2]*m*-benzo[4]pyrrole (**2**), 10α,20β- and 10α,20α- bis(4-nitrophenyl)-calix[4]pyrroles **3** and **4,** respectively**,** were found to exhibit various organocatalytic activities in the diastereoselective vinylogous addition reaction of 2-trimethylsilyloxyfuran (TMSOF, **7**) to aldehydes. The γ-hydroxybutenolide products are obtained in fairly good yields and with moderate diastereoselectivity. The structures of the catalysts, as well as the reaction conditions, strongly influence the efficiency of the reaction.

## Introduction

Calixpyrroles are macrocyclic compounds capable of binding anions and neutral molecules by means of multiple hydrogen bonds with their pyrrole NH units [1,2,3]. Calix[4]pyrrole (**1**) is the simplest member of this class of receptors. As hydrogen bond donors, calixpyrroles have a potential to behave as organocatalysts in a way similar to that reported for taddols [4,5], binols [6,7,8,9], urea and thiourea derivatives [10,11,12,13,14,15,16]. Very recently we reported the first example (and proof) of this property for the hetero-Diels-Alder reaction of Danishefsky’s diene with *p*-nitrobenzaldehyde organocatalysed by 10α,20β-bis(4-nitrophenyl)-calix[4]pyrrole (**3**) [17]. Moreover, we have recently shown that the 10α,20α-isomer **4** can selectively recognize topologically different dihydroxyaphthalene oxoanions and can be used to achieve regioselective alkylations and acylation reactions [18].


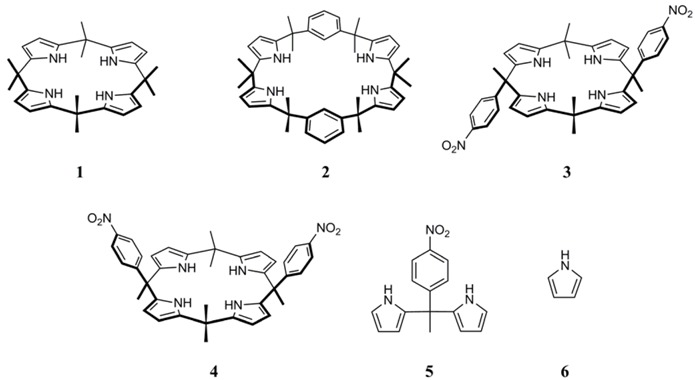


During our studies on the development of efficient organocatalysts for the addition of 2-trimethyl-silyloxyfuran (TMSOF, **7**) to aldehydes **8** we found that *N,N'*-bis[3-(trifluoromethyl)phenyl] urea activates carbonyl compounds through hydrogen-bonding interactions, and accelerates these vinylogous aldol reactions [19].

The products of this versatile carbon-carbon bond forming reaction are butenolide-like compounds which represent a substructure of a more complex assembly found in numerous biologically important natural and synthetic products [20,21,22,23]. The diastereoselective addition of variously substituted furan-based silyloxydiene synthons to a variety of achiral aldehydes and acetals using Lewis acids as catalysts has been reported in a number of papers [24,25,26,27,28,29,30]. MacMillan described the first enantioselective organocatalytic 1,4-addition of TMSOF to unsaturated aldehydes with high enantioselectivities [31]. However, our recent paper [19] describes the only example reported to date of an “organocatalyzed” aldol addition reaction of TMSOF to aldehydes.

**Scheme 1:**
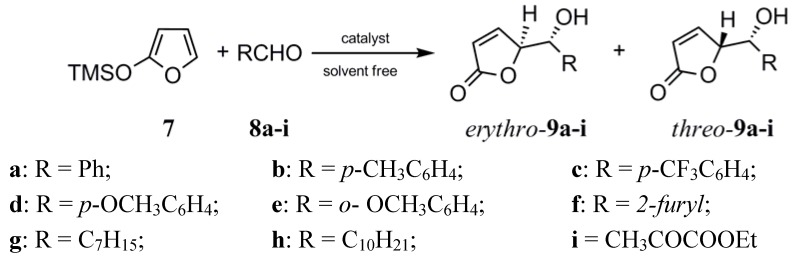
Diastereoselective addition reaction of TMSOF to aldehydes.

As part of our continuing research in this area, here we report a synthetic approach to compounds of type **9**, in which several calixpyrrole derivatives were used as organocatalysts for the first time.

## Results and Discussion

We selected the reaction of TMSOF (**7**) with benzaldehyde (**8a**) as an initial test of the catalytic properties of the calixpyrroles **1**-**4**. Compared to calix[4]pyrrole (**1**), receptors **2**, **3**, and **4** exhibit various selectivity towards the binding of anions, and in many instances they are stronger complexing agents [32,33]. To the extent that this feature arises from an enhanced ability to act as hydrogen bond donors**,** macrocycles **2**-**4** could be expected to be superior to **1** as organocatalysts. Macrocycles **3** and **4** also provide a means to explore the role of the stereochemically different substitution at two *meso*-positions. Dipyrromethane **5** and pyrrole **6** were also tested as catalysts because they represent structural units contained in **1-4**.

In an initial screening, the reactions were carried out at room temperature (25 °C) for 24 h, with 20% mol of the catalyst, based on TMSOF (0.5 mmol), without added solvent, the liquid benzaldehyde (2.5 mmol) being both the reactant and the solvent [19]. The silylated aldols were directly deprotected using Carreira’s procedure [34] to give the aldols *erythro-***9a** and *threo*-**9a**. All reactions (including those described later in this paper) were repeated at least three times, and the yields were found to be reproducible within 5%. In all cases, the catalysts were found to be unaffected at the end of the reaction and their recovery by flash chromatography was a viable option which was undertaken in some cases. The experimental results are summarized in Table 1.

**Table 1:** Results for the screening of catalysts **1**-**6** in the aldol addition of **7** to **8a**^ [a]^.

Entry	Catalyst	Yield (%) ^[b]^	erythro/threo^ [c]^
1	-	0	-
2	**1**	45	70/30
3	**2**	63	80/20
4	**3**	12	70/30
5	**4**	30	70/30
6	**5**	16	60/40
7	**6**	25	70/30
8	**6**	0	-

^[a]^ Reaction conditions: TMSOF (0.5 mmol), benzaldehyde (2.5 mmol), 25°C, 24 h. The catalyst was 20 mol% (based on TMSOF) in all cases with the exception of entry 8, where 80 mol% catalyst was used; [b] Combined isolated yield of *erythro*-**9a** and *threo*-**9a**; [c] The diastereoisomeric ratio (*erythro/threo*) was determined by ^1^H-NMR analysis of the crude product according to literature data [35].

In the absence of catalysts (Table 1, entry 1), no reaction is observed. Calix[4]pyrrole (**1**) (Table 1, entry 2) acts as a Lewis acid and enhances the reactivity of **8a** towards TMSOF **7**, affording the adducts **9a** in 45% yield with a good diastereomeric ratio. Macrocycles **3** and **4** (Table 1, entries 4-5) were found to be less active than the parent compound **1**, although they are stronger hydrogen-bond donors than **1** towards anions (*e.g.* fluoride). We believe this reduced activity to be due to the steric crowding of the macroring by the *p*-nitrophenyl units. Macrocycle **2** (Table 1, entry 3) was the most effective catalyst, probably because it is also the best anion ligand and it also lacks steric barriers at the *meso*-positions. When used in a 20% molar ratio, pyrrole **6** (Table 1, entry 7) was almost as active as macrocycle **4**, but surprisingly it was totally inactive (no reaction was observed) at an 80% molar ratio (Table 1, entry 8, to account for the presence of four pyrrole units in the macrocycles under scrutiny). A pattern emerges, whereby the most active catalysts also exhibit the highest diastereomeric ratios.

**Table 2:** Effects of reaction conditions on the reaction of **7** with **8a** catalyzed by **1** and **2.**

Entry	Catalyst (mol%)	°C/h	Yield (%) ^[b]^	erythro/threo ^[c]^
1^[a]^	**1** (20)	25°/48	82	70/30
2^[a]^	**1** (20)	25°/72	18	70/30
3^[a]^	**1** (20)	40°/24	7	70/30
4^[a]^	**2** (20)	25°/48	74	70/30
5^[a]^	**2** (20)	40°/48	34	78/22

^[a]^ Reaction conditions: TMSOF (0.5 mmol), benzaldehyde (2.5 mmol). The catalyst (mol%) is based on TMSOF; ^[b]^ Combined isolated yield of *erythro*-**9a** and *threo*-**9a**; ^[c] ^The diastereoisomeric ratio (*erythro/threo*) was determined by ^1^H-NMR analysis on the crude product according to literature data [35].

We then investigated the effects of changes in the reaction conditions in order to improve the activity of the best catalysts **1** and **2** (see Table 2). With catalyst **1**, compared to the initially tested conditions (Table 1, entry 2), extending the reaction time to 48 h increased the yield to 82% (Table 2, entry 1), but longer periods (Table 2, entry 2) gave lower yields, probably because of a retro-aldol reaction. At a higher temperature, (Table 2, entry 3) the yields decreased, probably because of a reduced binding between the catalyst and the aldehyde. 

With catalyst **2**, extending the reaction time from 24 (Table 1, entry 3) to 48 h (Table 2, entry 4) increased the yield of adducts **9a** from 63 to 74%, but with no improvement in the diastereomeric ratio. At a higher temperature the yields of **9a** were reduced (Table 2, entry 5).

Encouraged by the results obtained for the reaction with benzaldehyde, we tested the scope and limitations of this reaction with different aldehydes in the best conditions (see Table 2, entries 1 and 4) found for catalysts **1** and **2**. The results are reported in Table 3. Entry 8 shows that these catalysts are also effective when the carbonyl unit is that of an activated ester. For the different aldehydes the yields of the reactions are generally consistent with the reactivities observed with other hydrogen-bonding organocatalysts. Thus, for example, **8c** is more reactive than **8b**, and aliphatic aldehydes are generally less reactive than the aromatic ones.

However, there are significant differences in the yields for several aldehydes, depending on the catalyst used. The most remarkable of these are entries 3, 4, 5, and 7. Moreover, catalyst **2** appears more selective than **1** towards some different aldehydes (e.g*.* see entries 2 and 4 in Table 3). 

There are a number of organocatalysts for the reactions described here that are more readily accessible than the calixpyrroles used in this study [19]. However, the observed selectivity of the calixpyrroles towards different substrates is a feature of particular interest in view of future applications that involve the combination of molecular recognition with organocatalytic properties. This concept has been highlighted as an appealing goal in supramolecular catalytic systems [36].

**Table 3:** Aldol reaction of TMSOF (**7**) with carbonyl compounds **8b-i** catalyzed by **1** or **2**^[a]^.

Entry	Substrate	Yield (%)^[b]^ with 1	Yield (%)^[b]^ with 2	*erythro*/*threo*^[c]^ with 1	*erythro*/*threo*^[c]^ with 2
1	*p*-CH_3_-C_6_H_4_CHO **(8b)**	40	40	80/20	80/20
2	*p*-CF_3_-C_6_H_4_ CHO **(8c)**	58	50	70/30	70/30
3	*p*-OCH_3_-C_6_H_4_ CHO **(8d)**	25	4	80/20	-
4	*o*-OCH_3_-C_6_H_4_ CHO **(8e)**	15	0	70/30	-
5	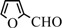 **(8f)**	70	56	70/30	70/30
6	C_7_H_15_ CHO **(8g)**	30	40	70/30	70/30
7	C_10_H_21_ CHO **(8h)**	16	30	70/30	70/30
8	CH_3_COCOOEt **(8i)**	63	63	70/30	70/30

^[a]^ Reaction conditions: TMSOF (0.5 mmol), aldehyde (2.5 mmol), 25 °C, 48h. The catalyst (20 mol%) is based on TMSOF; ^[b]^ Combined isolated yield of *erythro*-**9a** and *threo*-**9a**; ^[c] ^The diastereoisomeric ratio (*erythro/threo*) was determined by ^1^H-NMR analysis on the crude product according to literature data [35].

## Experimental

### General

All chemicals were standard reagent grade and were used without further purification. All air-sensitive and/or moisture-sensitive reactions were conducted under an inert atmosphere. Thin-layer chromatography was performed on Merck Kiesegel 60 (0.25 mm) eluting with the solvents indicated, visualized by a 254 nm UV lamp or aqueous ceric sulfate solution followed by heating. Column chromatography was carried out using silica gel 60 (70-230 mesh, Merck). NMR spectra were recorded on a Bruker DRX 400 (400.13 MHz for ^1^H and 100.03 for ^13^C). 

### General Procedure for the Organocatalytic Addition of TMSOF to Aldehydes

A mixture of catalyst **1** or **2** (0.1 mmol) and the appropriate aldehyde **8a-i** (2.5 mmol) was stirred at room temperature for 15 minutes. Then, TMSOF (**7**, 84 μL, 0.5 mmol) was added dropwise and the resulting solution was stirred at room temperature for the time reported in Table 1, Table 2, Table 3. The progress of the reaction was monitored by TLC. Upon completion, the mixture was cooled at –30°C and THF (0.5 mL) and TFA (0.2 mL) were added. The solution was then warmed to r.t. and stirred for 1 h after which the desilylation was complete. The reaction mixture was diluted with ethyl acetate and a saturated aqueous solution of NaHCO_3_ (2 mL) was added dropwise. The mixture was stirred until the evolution of gas ceased (30 min), the organic layer was then separated, washed with brine, dried over MgSO_4_, and concentrated *in vacuo*. The crude mixture was purified by silica gel column chromatography (eluent: hexane/ethyl acetate) to afford a mixture of the corresponding diastereomeric butenolides **9a-i**. The products are all known compounds identified by their spectroscopic data and comparison with literature values [19,37,38]. The diastereomeric ratio was determined by ^1^H-NMR analysis.

## Conclusions

The results described here prove that the exploration of the organocatalytic properties of calixpyrrole derivatives, which we started just over one year ago, is still rich in novel possibilities. Thus, the calixpyrroles **1-4** displayed varied organocatalytic activities in the diastereoselective vinylogous addition reaction of 2-trimethylsilyloxyfuran to aldehydes, affording γ-hydroxybutenolide products. The fairly good yields and moderate diastereoselectivities obtained were comparable to those previously observed with other organocatalysts [19]. We are actively involved in testing the activity of various calixpyrroles as organocatalysts in other different reactions.
